# Pharmacological Effects of Polyphenol Phytochemicals on the JAK-STAT Signaling Pathway

**DOI:** 10.3389/fphar.2021.716672

**Published:** 2021-09-03

**Authors:** Qianqian Yin, Longyun Wang, Haiyang Yu, Daquan Chen, Wenwei Zhu, Changgang Sun

**Affiliations:** ^1^ College of Traditional Chinese Medicine, Shandong University of Traditional Chinese Medicine, Jinan, China; ^2^ State Key Laboratory of Component-based Chinese Medicine, Tianjin University of Traditional Chinese Medicine, Tianjin, China; ^3^ School of Pharmacy, Yantai University, Yantai, China; ^4^ Yueyang Hospital of Integrated Traditional Chinese and Western Medicine, Shanghai University of Traditional Chinese Medicine, Shanghai, China; ^5^ Department of Oncology, Weifang Traditional Chinese Hospital, Weifang, China; ^6^ Qingdao Academy of Chinese Medical Sciences, Shandong University of Traditional Chinese Medicine, Qingdao, China

**Keywords:** JAK, STAT, signaling pathway, polyphenols, phytochemicals

## Abstract

The JAK-STAT signaling pathway is a common pathway of many cytokine signal transductions, closely related to cell proliferation, apoptosis, differentiation, and inflammatory response. It is essential for inhibiting the inflammatory response, initiating innate immunity, and coordinating adaptive immune mechanisms. Owing to the nature of this pathway and its potential cross-epitopes with multiple alternative pathways, the long-term efficacy of monotherapy-based adaptive targeting therapy is limited, and the majority of drugs targeting STATs are still in the preclinical phase. Meanwhile, curcumin, quercetin, and several kinds of plant polyphenol chemicals play roles in multiple sites of the JAK-STAT pathway to suppress abnormal activation. Polyphenol compounds have shown remarkable effects by acting on the JAK-STAT pathway in anti-inflammatory, antitumor, and cardiovascular disease control. This review summarizes the pharmacological effects of more than 20 kinds of phytochemicals on JAK-STAT signaling pathway according to the chemical structure of polyphenolic phytochemicals.

## Introduction

The Janus kinase (JAK)/signal transducer and activator of transcription (STAT) signaling pathway is a simple membrane-to-nucleus signaling pathway (O’Shea et al., 2015), used by quantities of cytokines, growth factors, and interferons, and extracellular factors also regulate gene expression through this pathway. It is the fundamental paradigm for cells to sense environmental cues and interpret these signals to regulate cell growth and differentiation, and is extensively involved in cell differentiation, proliferation, apoptosis, and inflammatory response processes (Yang et al., 2020b). Numerous experiments have shown the JAK-STAT pathway to be abnormally activated in rheumatoid arthritis (RA), Parkinson’s disease, multiple sclerosis, inflammatory bowel disease, sepsis, and development of tumors (Westhovens et al., 2017). Because of the role of the JAK-STAT signals in the universality of autoimmune diseases and malignancies, targeting the JAK-STAT pathway to initiate a congenital immune response, coordinated adaptive immunity, and to inhibit inflammation is crucial.

The intracellular components of the cytokine signaling pathway, especially the JAK family of non-receptor tyrosine kinases that transmit signals, can be used as key targets to inhibit the effects of various cytokines. A variety of JAK inhibitors, such as baricitinibde, tofacitinib, and ruxolitinib, have been approved by the United States Food and Drug Administration for the treatment of RA and psoriatic arthritis (PSA) (T Virtanen et al., 2019). However, multiple adverse reactions of JAK inhibitors have also been reported. All the JAK inhibitors, except filgotinib, reduce natural killer cells. Almost all JAK inhibitors increase the incidence of herpes zoster, while baricitinib and upadacitinib increase the risk of venous thromboembolism (Reddy and Cohen, 2020). Although STATs are abnormally activated in malignancies and immune-related diseases, it is more challenging as a therapeutic target than JAKs because of its lack of enzyme activity, bioavailability, *in vivo* efficacy, and selectivity (Schwartz et al., 2016). STAT3 is the important member of the STAT family that has been the focus of therapeutic attempts (except for Fludarabine, which targets STAT1), but the majority of drugs targeting STATs are still in the preclinical phase. Three therapeutic strategies are currently being used by targeting the upstream and downstream STAT activation: 1) inhibitory peptides, which sequester STATs from upstream receptors and kinases, 2) small-molecule inhibitors, which impede STAT activation and/or function, and 3) decoy oligonucleotides, which sequester STATs away from genomic binding sites (Hayakawa et al., 2013).

Phytochemicals are widely used in the treatment of diseases due to their multi-target effects, among which polyphenolic phytochemicals have been frequently reported to interfere with cell proliferation, apoptosis, differentiation and inflammation by acting on multiple sites in the JAK-STAT pathway. Polyphenols are a class of complex secondary metabolites with multiple phenolic hydroxyl groups, which are commonly found in tea, fruits, and vegetable substrates. According to the number of phenolic rings they contain, they can be roughly divided into flavonoids (flavones, flavonols, isoflavones, anthocyanidins, flavanones, flavanols, chalcones, and biflavones), phenolic acids, stilbenes, lignans and some compounds with complex parent nuclei, such as Curcumin and Wedelolactone (Yi et al., 2019). Most polyphenol phytochemicals have antioxidant, anti-inflammatory, and antitumor effects, and inhibit cardiovascular diseases (Sun et al., 2019a). Multiple polyphenolic phytochemicals inhibit abnormal activation and reduce resistance to targeted drugs by acting on multiple sites. In order to make a systematic summary of polyphenols phytochemicals, this paper reviews the pharmacological effects of more than 20 polyphenols on the JAK-STAT signaling pathway.

## JAK-STAT Signaling Pathway

### Composition and Transmission of JAK-STAT Signaling Pathway

#### JAK

JAKs are a family of nonreceptor tyrosine kinases in cells, consisting of four members: JAK1, JAK2, JAK3, and Tyk2 (Kurdi et al., 2012). The JAK protein family mediates the signal transduction of various hormones and cytokines, such as growth hormones, immune system regulators, and hematopoietic factors (Xin et al., 2020). The JAK protein family is structurally composed of seven JAK homology (JH) domains, the classical protein tyrosine kinase domain (JH1); the pseudokinase domain (JH2); the Src-homology type 2 (SH2) domain (JH3-JH5), and the FERM domain (JH6-JH7) (Garrido-Trigo and Salas, 2020). Although JAK3 is mainly expressed in hematopoietic cells, the other three members are thought to be universally expressed in tissues. JAK does not only phosphorylate the bound cytokine receptor but also phosphorylates multiple SH2 domain-specific signaling molecules (Agrawal et al., 2011).

#### STAT

The STAT family is a class of cytoplasmic proteins that can bind to the DNA in the regulatory region of target genes and are downstream targets of JAK (Xu et al., 2019). The STAT family consists of the following members: STAT1–STAT6; STAT5 includes STAT5A and STAT5B. The STAT proteins can be structurally divided into five main domains: N-terminal conserved sequence, DNA binding domain, SH3 domain, SH2 domain, and C-terminal transcriptional activation domain (Chang et al., 2018b). STATs bind to DNA regulatory elements, and direct the transcription of mRNAs or long noncoding RNAs.

#### Activation and Inhibition of the JAK-STAT Pathway

Under normal physiological conditions, the STAT protein exists in the cytoplasm in an inactive form or is briefly activated (Bodmer et al., 2020). A variety of cytokines attach to type I/II cytokine receptors to dimerize the receptors. The tyrosine residue on the receptor aggregates with JAK (Stokes et al., 2015). The surrounding JAKs are activated by phosphorylation of each other, and the activated JAK protein phosphorylates itself and these tyrosine residues, providing a docking site for the SH2 domain of the STAT protein (Ashrafizadeh et al., 2020). Phosphorylation induces the dimerization of STAT by the conserved SH2 domain, which then allows it to enter the nucleus via the α-5 and RAN nuclear input pathways (Ramalingam et al., 2017).

**FIGURE 1:**
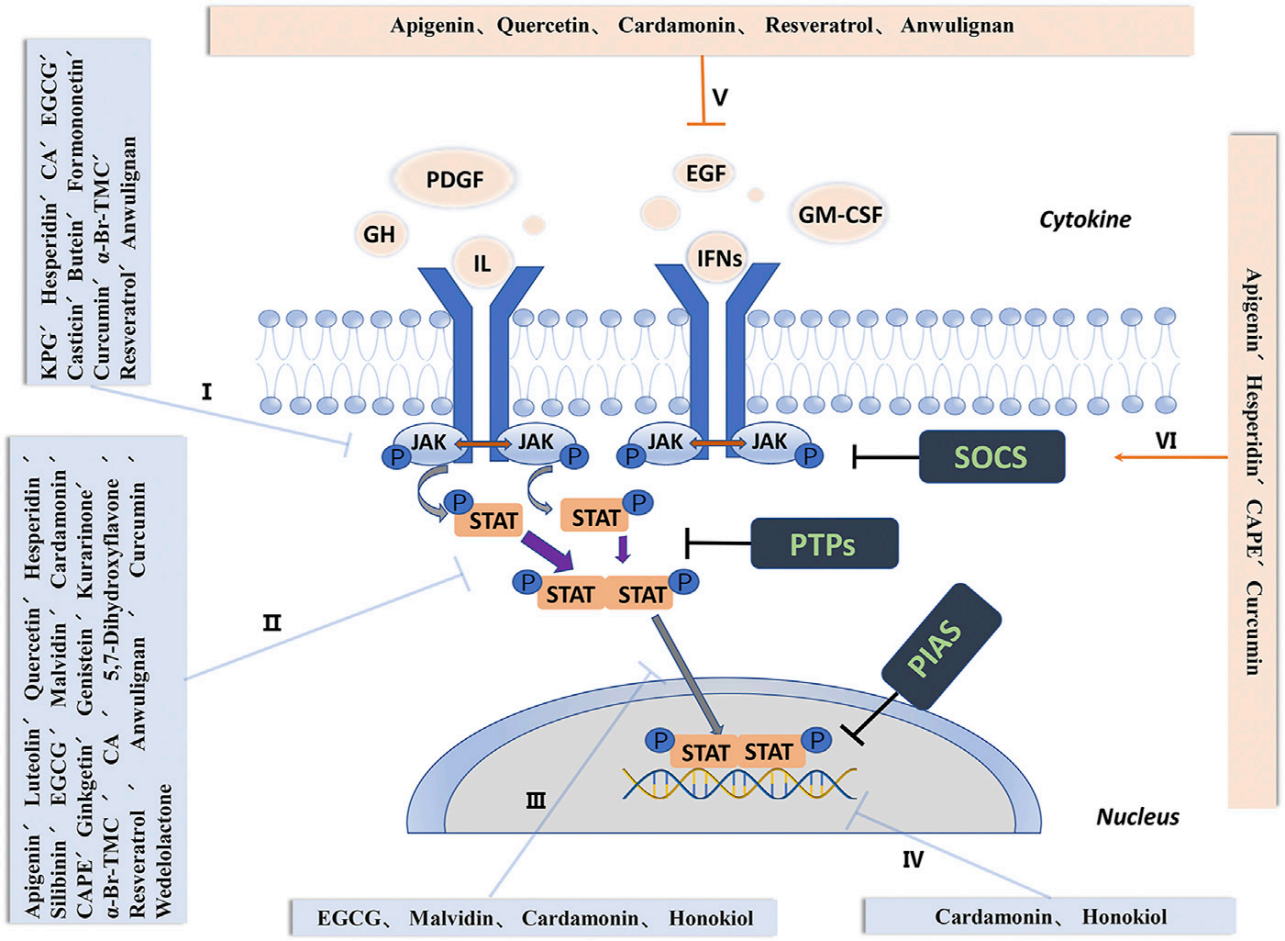
Activation and negative regulation of the JAK-STAT signaling pathway. I, Inhibit activation and phosphorylation of JAK. II, Inhibit the activation, phosphorylation and formation of dimers of STAT. III, Interfere with the transfer of STAT to the nucleus. IV, Interfere with the binding of STAT and DNA and regulate the expression of STAT target genes. V, Up-regulate the secretion and expression of negative feedback signal SOCS. VI, Inhibit the secretion of interleukin, interferon, tumor necrosis factor and binding to receptors. Abbreviations: GH, growth hormone; PDGF, platelet-derived growth factor; EGF, epidermal growth factor; IFNs, interferons; IL, interleukins; GM-CSF, granulocyte-macrophage colony-stimulating factor; JAK, janus kinase; STAT, signal transducer and activator of transcription; SOCS, suppressor of cytokine signaling; PTPs, protein tyrosine phosphatases; PIAS, protein inhibitors of activated STATs.

The JAK-STAT signaling pathway is negatively regulated by three endogenous inhibitors: suppressor of cytokine signaling (SOCS), PTPs, and the protein inhibitors of activated STATs (PIAS). SOCS inhibit JAK activity, promote proteasomal degradation of JAK, and compete with STATs to bind to the cytokine receptors (Byers et al., 2009). PTPs induces STAT inactivation either by dephosphorylation of STAT proteins or by targeting associated receptor kinase complexes. It has been proved that the PIAS protein family consists of at least five members, and structurally contains four common domains, including one N-terminal SAP domain. Different members of the PIAS family can reduce the transcriptional activity of STAT and inhibit the binding of STAT to DNA (Shuai, 2000) (Figure 1).

### Relationship Between JAK-STAT Mutation and Disease

In 2003, the first confirmed case of a mutation in the JAK-STAT pathway, a homozygous mutation in the STAT5B gene, was reported, resulting in IGF-1 deficiency and insensitivity to growth hormone. After birth, the patient had abnormal growth, facial deformities, and significantly reduced acid-labile subunit (Kofoed et al., 2003). Several diseases were subsequently found to be associated with known JAKs and STATs mutations, and JAK-STAT dysfunction is commonly attributed to Loss-of-function (LOF) and Gain-of-function (GOF) mutations that may trigger the disease (Villarino et al., 2020) (Table 1). It was proven that GOF mutations in JAK1, JAK2, and JAK3 are responsible for hematopoietic disorders such as essential thrombocytopenia, acute myeloid leukemia, or Hodgkin Lymphoma. STAT1 and STAT2 play an important role in antitumor immune responses. STAT3 and STAT5 are associated with tumor genesis and progression, and STAT3, in particular, is closely associated with tumor cell survival, immunosuppression, and persistent inflammation (Villarino et al., 2020).

**TABLE 1:** JAK-STAT mutations and resulting disorders.

Mutations of JAK		
**JAKs**	**Disease**	**References**
JAK1	Atypical mycobacterial	O'Shea et al. (2013)
	Solid neoplasms	Li and Chen (2021)
	Bronchopneumonia	He and Tian (2021)
	Liver inflammation	Daryadel et al. (2021)
	Rheumatoid Arthritis (RA)	Westhovens et al. (2017)
	Atopic dermatitis (AD)	Kim et al. (2020)
	Diabetic nephropathy (DN)	Tuttle et al. (2018)
JAK2	Lymphoma	Van Den Neste et al. (2018)
	Aorta aneurysm	Elf (2021)
	Esophageal squamous cell carcinoma (ESCC)	Li et al. (2021)
	Pulmonary hypertension (PAH)	Leopold (2021)
	Atopic dermatitis (AD)	Kim et al. (2020)
JAK3	Severe combined immunodeficiency (SCID)	O’Shea et al. (2013)
	Leukemia	Tefferi (2008)
TYK2	Immunodeficiency.	Casanova et al. (2012)
	Mycobacterium infection	Minegishi et al. (2006)
**Mutations of STAT**		
**STATs**	**Disease**	**References**
STAT1	Autoimmune disease (AID)	Patel et al. (2018)
	Chronic myelogenous leukemia (CML)	Noguchi et al. (2018)
	Candidiasis (CMC)	Zheng et al. (2015)
STAT2	Viral susceptibility	Moens et al. (2017)
	Ahronic myelogenous leukemia (CML)	Takahashi et al. (2018)
STAT3	Hyperimmunoglobulin E syndrome	Mohr et al. (2013)
	Leukemia	Barilà et al. (2020)
	Lymphoma	Reilley et al. (2018)
	Solid neoplasms	Yuan et al. (2020)
	Inflammation	Ni et al. (2017)
	Sjogren syndrome (SS)	Aota et al. (2021)
STAT4	Sjogren syndrome (SS)	Korman et al. (2008)
	Kaposis sarcoma (KS)	Aavikko et al. (2015)
	Rheumatoid arthritis (RA)	Yang et al. (2020a)
	Systemic lupus erythematosus (SLE)	Patel et al. (2018)
	Systemic sclerosis (SSC)	Xu et al. (2016)
STAT5A/B	Leukemia	Dumas et al. (2019)
	Lymphoma	Zhang et al. (2019)
	Systemic lupus erythematosus (SLE)	Wu et al. (2017)
	Eosinophilia	Kagami et al. (2000)
	Solid neoplasms	Li et al. (2020b)
	Inflammation	Surbek et al. (2021)
STAT6	Lymphoma	Yang et al. (2009)
	Allergic inflammation	Ghaffar et al. (2000)

## Pharmacological Effects of Polyphenol Phytochemicals on the JAK-STAT Signaling Pathway

### Flavonoids

Flavonoids are polyphenols found in plants with a broad spectrum of biological activities (Guan and Liu, 2016). They widely exist in fruits, vegetables, tea, cereals, and other plants, and belong to plant secondary metabolites. Flavonoids generally refer to a series of compounds consisting of two benzene rings (A and B rings) with phenolic hydroxyl groups connected to each other through three central carbon atoms (Li et al., 2020a). According to the oxidation degree of the central three-carbon chain (whether the 2,3-position is a double bond, whether the 4-position is a carbonyl group), the connection position of the B-ring (2-or 3-position) and whether the three-carbon chain forms a ring, flavonoids can be divided into the following categories: Compounds with 2,3-position double bond and 4-position carbonyl group are flavones; compounds with hydrogenated 2,3-position are flavanonols; compounds with 4-position carbonyl group reduction are flavanols; compounds with B ring at 3-position are isoflavones; and compounds with C ring opening ring are chalcones (Khokra et al., 2016). In addition, there are still some flavonoids have a unique mother nucleus structure, such as xanthones, aurones, pterocarpins. In plants, most of them combine with sugars to form glycosides, and some exist in free form. A number of *in vitro* studies have shown that flavonoids can inhibit the abnormal activation of the JAK-STAT pathway, and have outstanding medicinal value with anti-inflammatory, antioxidant, anticancer, antidepressant and antimicrobial properties, and are often used to prevent and treat cardiovascular and cerebrovascular diseases, for immune regulation, and liver protection. In addition, flavonoids have similar effects to phytoestrogens.

#### Flavones

##### Apigenin

Apigenin is a plant-derived flavonoid that is abundant in celery, plantain seed, and is effective in the prevention and treatment of prostate cancer, and in inhibiting tumorigenesis and angiogenesis in melanoma, breast, skin, and colon cancers. Apigenin reduces p-JAK1/2 and p-STAT3 in breast cancer (BT-474) cells, and demonstrates anticancer activity by inhibiting JAK-STAT (Ozbey et al., 2018). Programmed death in colon cancer cells can be induced by inhibition of STAT3 phosphorylation, thereby downregulating the antiapoptotic proteins Bcl-XL and Mcl-1 (Maeda et al., 2018). Apigenin can suppress the levels of IL-4, IL-5, IL-13, and IFN-g in Ovalbumin-Induced Allergic Rhinitis mouses Nasal lavage fluid (NLF) and inhibit the expression of p-STAT6 and SOCS1 (Chen et al., 2020).

##### Luteolin

Luteolin is a secondary plant metabolite that belongs to flavonoids and is widely distributed in honeysuckle, Perilla, pepper and other herbs and vegetables, mainly in the form of glycosides in plants; luteolin inhibits epithelial-mesenchymal transition (EMT) and matrix metalloproteinase (MMP) secretion likely through deactivation of STAT3 signaling, to directly reduce the phosphorylation level of STAT3 in cancerous cells of pancreas, and inhibits IL-6 to reduce the invasion of pancreatic cancer cells (Huang et al., 2015). Luteolin has a synergistic effect with paclitaxel, which can effectively reduce p-STAT3 in mouse breast cancer cells and induce breast tumor regression in mice injected subcutaneously with MDA-MB-231 cells together with paclitaxel (Yang et al., 2014). Luteolin also disrupts Hsp90 and STAT3 expression in gastric cancer cells. Luteolin activates SHP-1, a protein tyrosine phosphatase that dephosphorylates STAT3, and reduces the expression of its target genes, which “shut down” the regulation of target genes by inhibiting nuclear accumulation of STAT proteins, thereby demonstrating the efficacy of luteolin in inducing tumor growth inhibition *in vivo* (Song et al., 2017a).

#### Flavonols

##### Kaempferol 7-O-β-D-glucoside

KPG is a natural flavonol isolated from *Hostaplantaginea (Lam.) Aschers* with anti-cancer and anti-inflammatory effects (Tian et al., 2005), which inhibits p-STAT1 and p-STAT3 by inactivating p-JAK1/2 in lipopolysaccharide-induced inflammation. Inactivation of STAT1/3 in macrophages inhibits the expression of lipopolysaccharide (LPS)-induced pro-inflammatory mediators, and attenuates the JAK-STAT signaling pathway in macrophages (Lee et al., 2018).

##### Quercetin

Quercetin is a natural flavonoid compound that is widely found in buckwheat, hawthorn and sea-buckthorn, and has various pharmacological effects such as antioxidant, anti-aging, and anti-inflammatory effects (Wang et al., 2021). Muthian et al. treated activated T cells with quercetin to block IL-12-induced JAK2, Tyk2, STAT3, and STAT4 tyrosine phosphorylation, thereby reducing IL-12-induced T cell proliferation and Th1 differentiation, to improve experimental autoimmune encephalomyelitis (EAE) (Muthian and Bright, 2004). Senggunprai et al. found that quercetin combined with Epigallocatechin-3-gallate (EGCG) suppressed STAT1 and STAT3 phosphorylation in human holangiocarcinoma (CCA) cells (Senggunprai et al., 2014). In peripheral arterial disease, quercetin inhibits the decline of miR-216a and blocks the JAK2-STAT3 and PI3K/Akt pathways, thereby inhibiting HMEC-1 cell survival, migration, and tubulation (Wang et al., 2020).

#### Flavanones

##### Hesperidin and 5,7,3ʹ-Triacetyl Hesperetin

Hesperidin is mainly derived from the immature citrus fruits of the family Rutin and belongs to one of the dihydroflavones (Zhao et al., 2021). As natural products, hesperidin and TAHP possess extensive pharmacological properties, such as neuroprotective, and anti-inflammatory properties, inhibit the secretion of TNF-α and IL-6 and reduce the phosphorylation of NF-κB; hesperidin in combination with cinnamaldehyde significantly decreased p-JAK2 and p-STAT3 and significantly increased the expression of SOCS3 protein in ulcerative colitis (Elhennawy et al., 2021).

TAHP is a structural derivative of hesperidin. In a rat model of adjuvant arthritis, isomolar TAHP showed more effective anti-inflammatory activity than its parent compound hesperidin, and TAHP treatment reduced the expression of STAT3 and JAK2 genes in synovial tissue to varying extents (Ren et al., 2013).

##### Silibinin

Silibinin is a flavanone produced from the Milk Thistle, which has anticancer and hepatoprotective effects. When used in combination with traditional chemotherapy, the side effects of chemotherapy drugs, such as nephrotoxicity, gastrointestinal toxicity, and cardiotoxicity, can be reduced, thereby preventing or even reversing chemotherapeutic resistance (Bosch-Barrera et al., 2017). Silibinin also inhibits STAT3 phosphorylation, thereby reducing its transcription activity in ethyl carbamate -induced lung cancer cells in mice (Tyagi et al., 2009). It also inhibits the constitutive activation and apoptosis induction of STAT3 in prostate cancer cells. Compared with silibinin alone, JAK1 inhibitors combined with silibinin also lead to complete reduction of STAT3 phosphorylation at Tyr705, activation of caspase, and apoptosis of prostate cancer cells (Agarwal et al., 2007).

#### Flavanols

##### Epigallocatechin-3-Gallate

Natural Flavanols include flavan-3-ols, flavan-3,4-diols, and flavan-4-ols (Zakaryan et al., 2017). The derivatives of Flavan-3-ols are called catechins. EGCG is a catechin monomer isolated from green tea. STAT3 participates in adipocyte secretome-mediated Paracrine regulation of Human MDA-MB-231 (TNBC-derived) cell invasive phenotype, EGCG inhibits p-STAT3 (Gonzalez Suarez et al., 2021). And in IFN-γ-stimulated human oral cancer cells, p-JAK1 and p-JAK2 are inhibited by EGCG, and EGCG inhibits STAT1 translocation to the nucleus (Cheng et al., 2010).

#### Isoflavones

##### Formononetin

FN is a natural isoflavone found in plants, including *Astragalus membranaceus,* Clover, *Glycyrrhiza glabrifolia, Radix puerariae, and Ryegrass multiflorum*. It is considered a typical phytoestrogen and has many biological functions, including neuroprotective, cardiac protection, anticancer, insulin resistance, and immunity regulation (Li et al., 2014). FN reduced JAK1-STAT1 phosphorylation and promoted myoblast differentiation (Soundharrajan et al., 2019). FN promotes apoptosis and inhibits cell proliferation in colorectal cancer by inhibition of cyclin D1 and MMP2/9 expression via p-STAT3 inactivation (Tay et al., 2019). FN also inhibited the phosphorylation and gene expression levels of JAK2 and STAT in HUVECs exposed to high glucose concentrations, showing great potential in the treatment of vascular complications of diabetes (Zhou et al., 2019). FT showed a significant anticancer effect in multiple myeloma (MM), which may be mainly through reactive oxygen species (ROS) indirectly down-regulating p-JAK1/2, inhibiting the activation of constitutive p-STAT3, and reducing the binding ability of STAT3/5 to DNA and the translocation of p-STAT3/5 to the nucleus (Kim et al., 2018).

##### Genistein

Genistein is a flavonoid that exists in beans and some Chinese herbal medicines, which has a variety of biological effects (Luo et al., 2018). Genistein inhibits GATA-3 and STAT6 and increases the production of T-box transcription factor (T-bet), thereby reducing Th2 type cytokines, increasing Th1 type cytokines, regulating Th1/Th2 response, and alleviating airway inflammation caused by ovalbumin (OVA) (Gao et al., 2012). In Murine J774 macrophages, Genistein inhibits the activation of STAT1, another important transcription factor of inducible nitric oxide synthase (iNOS), In the macrophages of Murine J774 macrophages, Genistein inhibits the activation of STAT1, another important transcription factor of inducible nitric oxide synthase (iNOS), explaining the pharmacological effect of Genistein as an anti-inflammatory compound (Hämäläinen et al., 2007).

#### Anthocyanidins

##### Malvidin

Malvidin, a major anthocyanin in blueberries, is involved in the inhibition of inflammation-related mediators in inflammatory diseases and has become a promising anticancer drug because of its powerful antioxidant and antiproliferative effects (Bastin et al., 2021). Treatment with blueberry and malvidin alone or in combination, significantly downregulated the expression of the phosphorylated forms of JAK2 and STAT3. Furthermore, it inhibits nuclear translocation of STAT3, prevents proliferation, induces apoptosis of oral cancer cells *in vitro* and *in vivo* (Baba et al., 2017).

#### Chalcones

##### α-Bromo-2′,3,4,4′-Tetramethoxychalcone

Chalcone is a precursor of flavonoids and isoflavones and is found in a variety of fruits, vegetables, and spices, and occurs in nature in many conjugated forms. It has antidiabetic, antitumor, antihypertensive, antiretroviral, anti-inflammatory, antihistamine, antioxidant, anti-malarial, and other pharmacological properties (Mahapatra et al., 2015).

α-Br-TMC inhibited both JAK2 and STAT5 phosphorylation and altered the mobility of STAT5A/B proteins in SDS-PAGE in the IL-3-stimulated Ba/F3 cells and its oncogenic derivative Ba/F3-1*6. The tumor suppressor gene CIS and the oncogene c-Myc are the target genes of STAT5, α-Br-TMC down-regulated c-Myc and up-regulated CIS in Ba/F3-1*6 cells, therefore, α-Br-TMC is expected to be used in the treatment and intervention of STAT5 related malignant tumors in the future (Pinz et al., 2014).

##### 3,4,2′,4′-Tetrahydroxychalcone (Butein)

Butein is a flavonoid isolated from the bark of the sufa tree, which is believed to have hypotensive, antioxidant, anticancer, antidiabetic, and neuroprotective effects (Padmavathi et al., 2017). In human multiple myeloma (MM) cells, constitutive and IL-6 inducible STAT3 activation are inhibited by inhibiting tyrosine kinases such as JAK1 and JAK2. In addition, Butein downregulates the expression of STAT3 target genes such as Bcl-2 and Mcl-1, leading to increased apoptosis levels (Pandey et al., 2009).

##### Bavachin

Bavachin is a phytoestrogen found in the seeds of *Psoralea*. Bavachin inhibits the activation of STAT3 in MM cells and controls the proliferation of MM cells without cytotoxicity to normal cells (Takeda et al., 2018). In a recent study, IL-6-induced STAT3 activity was inhibited by Bavachin (Lee et al., 2012).

##### Cardamonin

Cardamonin is a chalcone found in *Zingiberaceae, Asteraceae, Pinaceae, Labiaceae*, and other plants and is closely related to antibacterial, anticancer, and other effects. Cardamonin inhibits the activation of ERK1/2 and STAT1-4 and reduces nitric oxide production by targeting the JAK-STAT pathway (Takahashi et al., 2011). Treatment with Cardamonin reduces the secretion of IL-1β and TNF-α in patients with recurrent colitis and colitis-associated tumors and inhibits cell viability and the production of inflammatory cytokines in colorectal cancer cells *in vitro*. In tumor cells, the inhibitory effect of Cardamonin on cell proliferation is closely related to reduced p-STAT (Hou et al., 2019). A study of the treatment of glioblastoma stem cells with Cardamonin found that the activation of STAT3 and downstream regulatory genes of STAT3 were inhibited by Cardamonin. In addition, Cardamonin inhibits the dimerization and nuclear migration of STAT3 in CD133+ GSCs (Wu et al., 2015).

#### 5,7-Dihydroxyflavone

5,7-Dihydroxyflavone is a natural flavonoid found in propolis, honey, and carnolium (Xie et al., 2011). 5,7-Dihydroxyflavone upregulated the expression of proapoptotic protein Bax, decreased the expression of antiapoptotic proteins Bcl-2, Mcl-1, and IAPS, decreased the phosphorylation levels of Akt and STAT3, and weakened the antiapoptotic signal, thereby promoting human liver cancer cells apoptosis (Zhang et al., 2013).

#### Biflavones

##### Ginkgetin

GK is a biological flavonoid compound extracted from *Ginkgo biloba* leaves and has anticancer, anti-inflammatory, antimicrobial, and neuroprotective activities. GK-treated preadipocytes inhibited the expression of peroxisomal proliferation-activated receptor γ (PPARγ) and CCAAT/enhancer binding protein α (C/EBPα) by inactivating STAT5. The above results indicate that GK can be used as anti-obesity drugs (Cho et al., 2019). GK mediates the dephosphorylation of STAT3 in Tyr705 and prevents it from entering the nucleus, thereby inhibiting STAT3-mediated gene expression, such as antiapoptotic Bcl-XL protein, thereby inhibiting the proliferation of prostate cancer DU-145 cells (Jeon et al., 2015). GK dose-dependently inhibits STAT3 phosphorylation and significantly reduces Survivin expression in osteosarcoma cells, thereby significantly inhibiting tumor growth (Xiong et al., 2016).

#### Ohers

##### Casticin

CST belongs to a group of polymethyl flavonoids obtained from the Vitex, showing a wide range of pharmacological properties, such as anticancer, anti-ulcerative colitis, and antioxidant properties (Song et al., 2017b). CST promotes apoptosis and suppresses p-JAK1 and p-JAK2 and the expression of the phosphorSrc kinase, thereby inhibiting the JAK-STAT pathway in tongue and oral squamous cell carcinomas (Lee et al., 2019).

##### Kurarinone

*Sophora flavescens*, an herbal medicine, contains quinoline alkaloids and flavonoids. Kurarinone has strong inhibitory effect on the immune response and effectively inhibits phosphorylation of STAT1/3 in mouse CD4+T cells (Kim et al., 2013). In addition, in CD4+T cells, the JAK1 and Tyk2-dependent phosphorylation of STAT1 and STAT4 was inhibited. Kurarinone also inhibits STAT6 phosphorylation induced by IL-4 and STAT5 phosphorylation induced by IL-2 in CD4+T cells.

#### Stilbenes

##### Resveratrol

Resveratrol is an astragalus compound found in berries, peanuts, and grapes. It is a candidate drug for adjuvant therapy in a variety of inflammatory diseases due to its anti-inflammatory and antioxidant activity (Pinheiro et al., 2019). Resveratrol can downregulate the expression of p-JAK, p-STAT, and inflammatory cytokines, and protects hippocampal neurons from brain ischemia reperfusion injury by regulating the JAK-ERK-STAT signaling pathway, thereby alleviating cognitive dysfunction (Chang et al., 2018a). Resveratrol can also reduce the expression levels of IL-6, TNF-α, IFN-γ, p-JAK1, and p-STAT3 (Tyr705) in the brain tissues of mice with autism, which is important for the treatment of neuroimmune dysfunction (Ahmad et al., 2018). Resveratrol inhibits IFN-γ-induced macrophage STAT1 transcription activity and IFN-γ-induced Tyr701 or Ser727 p-STAT1. It also inhibited IFN-γ-induced activation of JAK2 and extracellular signal-regulated kinases, of which JAK-2 was more sensitive, and blocked the JAK-STAT1 pathway to control the IFN-γ-activated macrophage inflammatory response (Chung et al., 2011).

#### Phenolic Acids

##### Caffeic Acidand Caffeic Acid Phenethyl Lester

CA and CAPE are phenolic compounds widely found in wine, tea, and coffee that have hepatoprotective, anti-inflammatory, antiviral, neuroprotective, and immunomodulatory effects (Çakır et al., 2011). In human renal carcinoma cells, CA and CAPE suppress tumor angiogenesis by inhibiting the activity of STAT3 and the expression of HIF-1α and Vascular Endothelial Growth Factor (VEGF) (Jung et al., 2007). In stroke-prone spontaneously hypertensive rats (SHRSP), CA inhibits angiotensin II (Ang II)-induced p-JAK2 and p-STAT1, and inhibit the generation of reactive oxygen species, thus partially blocking the JAK-STAT signaling pathway, thereby weakening Ang II-stimulated vascular smooth muscle cell proliferation reaction (Li et al., 2005). CAPE can protect the brain from the effects of toxicity of Chromium (VI) by reducing oxidative stress and inflammation in the brain of rats, enhancing antioxidant defense ability, and reversing the upregulation of Chromium (VI) on JAK2, STAT3 and SOCS3 in brain tissues (Mahmoud and Abd El-Twab, 2017).

#### Lignans

##### Anwulignan

Anwulignan is a monomer compound extracted from lignans in central China (Schroeder et al., 2020), showing a variety of pharmacological properties, including liver protection, antimicrobial, anti-inflammatory, regulation of neuronal survival, and anticancer effects. Anwulignan significantly inhibited the growth of non-small-cell lung carcinoma (NSCLC) cells and increased G1 cell cycle arrest. Anwulignan directly targets JAK1 *in vitro* to inhibit STAT3 phosphorylation in a dose-dependent manner, attenuate the JAK1-STAT3 pathway, and exert anticancer activity in NSCLC (Xie et al., 2021).

##### Honokiol

Honokiol is widely present in *Magnolia officinalis* and *Magnolia grandiflora*. It shows antiproliferation, proapoptosis, and regulation of cell cycle activity in the stomach, colon, pancreas, lung, and other solid tumors and leukemia by regulating a variety of carcinogenic targets (Bose et al., 2020). Honokiol inhibits both constitutive and inducible STAT3 activation and reduces the mRNA expression of STAT3 target genes (Bcl-2, cyclin D1, and survival) in myeloid leukemia cells in a concentration-dependent manner (Bi et al., 2015). It also inhibits STAT3 phosphorylation/activation in an Liver kinase B1 (LKB1)-dependent manner, preventing it from binding to the standard binding sites of Nanog, OC4, and SOX2 promoters (Sengupta et al., 2017). Huang et al. demonstrated that honokiol decreased the levels of phosphorylated JAK2 (p-JAK2) and phosphorylated STAT3 (p-STAT3) in oral cancer cells (Huang et al., 2016).

#### Others

##### Curcumin

Curcumin, a natural product of plant-derived polyphenols, can be isolated from the rhizome of *Curcuma*, and is a bioactive component with anti-inflammatory, antitumor, and antioxidative effects. It interferes with multiple signaling pathways that affect tumor proliferation, apoptosis, inflammation, and angiogenesis. In TNBS-induced intestinal inflammation rat models, curcumin can inhibit JAK-STAT pathway by enhancing SOCS-1 expression to achieve anti-inflammatory effects (Zhang et al., 2016). SOCS-1 inhibits JAK activity and promotes the proteasomal degradation of JAK. Induction of SOCS-1 led to a sharp downregulation of JAK2-STAT signaling and mRNA levels of target genes directly regulated by JAK2 (Ungureanu et al., 2002), and competition with STATs for binding to cytokine receptors, thus effectively inhibiting the JAK-STAT pathway and inhibiting pro-inflammatory responses. It can also dose-dependently inhibit the p-JAK2-p-STAT3 pathway to induce G0/G1 phase arrest and apoptosis, and inhibit the proliferation and migration of osteosarcoma cells (Sun et al., 2019b).

##### Wedelolactone

Wedelolactone is a coumarin found in *Wedelia calendulacea* and *Eclipta prostrata* that has anti-proteolytic, anti-inflammatory, anti-hepatotoxic, and anticancer properties owing to its multimolecular targets (Svrlanska et al., 2020). Wedelolactone significantly prolonged the duration of STAT1 tyrosine phosphorylation and prolonged the IFN-γ-induced STAT1 phosphorylation by inhibiting the T-cell protein tyrosine phosphatase (TCPTP)-dependent dephosphorylation of STAT1 and synergized with IFN-γ in inducing cell death among certain STAT1 expressing tumor cells (Chen et al., 2013) (Table 2).

**TABLE 2:** Pharmacological effects of polyphenol phytochemicals on the JAK-STAT signaling pathway.

Phytochemical name	Source of experimental evidence	Mechanism and effect	Effect	References
Apigenin	The human colon cancer cell lines HT29, DLD-1, COLO320, and HCT116	Inhibited STAT3 phosphorylation.	Inhibition of STAT3 phosphorylation, resulting in downregulation of antiapoptotic proteins Mcl-1 and Bcl-xL and induces programmed cell death in colon cancer cells.	Maeda et al. (2018)
	Adult male BALB/c mice	Decreased the levels of IL-4, IL-5, IL-13, and interferon-γ. Attenuated OVA-induced alterations in STAT6 and SOCS1 mRNA expressions.	It plays an anti-allergic role in allergic rhinitis (AR).	Chen et al. (2020)
Luteolin	Human pancreatic cancer cell lines	Inactivated p-STAT3 and downregulated STAT3 in a dose-dependent manner.	Luteolin inhibit pancreatic cancer cell invasion by inhibiting STAT3 signaling and secretion of EMT and MMP.	Huang et al. (2015)
	Human breast cancer MDA-MB-231 cells	Suppressed STAT3.	Luteolin enhances paclitaxel-induced apoptosis through inhibition of the STAT3 signaling pathway by mediating Fas expression and activation of caspases.	Yang et al. (2014)
	Gastric cancer (GC) cells	Inhibits STAT3 activation and dephosphorylated STAT3.	Luteolin selectively kills gastric cancer cells that are overactivated by STAT3, and these cells are usually drug-resistant.	Song et al. (2017a)
Kaempferol 7-O-β-D-glucoside (KPG)	The RAW 264.7 murine macrophage cell line	Inhibits the phosphorylation of JAK1 and JAK2.	KPG induces anti-inflammatory activity by blocking NF-κB, AP-1, and JAK-STAT signaling pathways in LPS-treated macrophages.	Lee et al. (2018)
Quercetin	SJL/J mice	Blocked IL-12 signaling.	*In vitro* treatment of activated T cells with quercetin blocks IL-12-induced tyrosine phosphorylation of JAK2, TYK2, STA T3, and STA T4, resulting in a decrease in IL-12-induced T cell proliferation and Th1 differentiation.	Muthian and Bright (2004)
	The human cholangiocarcinoma (CCA) cell lines	Inhibited IL-6 and IFN-γ and reduced p-STAT1, and STAT3 proteins in a dose-dependent manner.	Quercetin and EGCG are beneficial in inhibiting the JAK-STAT cascade in CCA cells.	Senggunprai et al. (2014)
	Human microvascular endothelial cells	Inhibition of miR-216a blocked the JAK2-STAT3 pathway.	Quercetin mainly inhibits survival, migration, and VEGF expression, and promotes apoptosis of HMEC-1 cells.	Wang et al. (2020)
Hesperidin and 5,7,3ʹ-triacetyl hesperetin (TAHP)	Adult male Wistar albino rats	Decreased p-JAK2 and p-STAT3 and increased SOCS3 protein level.	Cinnamaldehyde and hesperetin counteract TNBS-induced ulcerative colitis through modulation of the JAK2-STAT3-SOCS3 pathway.	Elhennawy et al. (2021)
	Male Sprague-dawley rats	Decreased STAT3 and JAK2.	TAHP play a crucial role in the pathogenesis of AA by regulating the production of proinflammatory cytokine IL-6 in serum and synovial tissue and inhibiting the over-activation of the JAK2-STAT3 signaling pathway.	Ren et al. (2013)
Silibinin	A/J male mice	Decreased the phosphorylation level of STAT3.	By inhibiting the activation of HIF-1α, NF-κB, and STAT3, lung tumor growth was inhibited.	Tyagi et al. (2009)
	Human prostate carcinoma DU145 cell	Inhibited constitutively active STAT3.	It effectively inhibited the constitutive activation and apoptosis induction of STAT3 in DU145 cells	Agarwal et al. (2007)
Epigallocatechin-3-gallate (EGCG)	Human MDA-MB-231 (TNBC-derived) cell	Inhibited p-STAT3.	EGCG prevented a STAT3-mediated paracrine oncogenic control of triple-negative breast cancer cell invasive phenotype	Gonzalez Suarez et al. (2021)
	Human oral cancer cells	Inhibited p-JAK1/2 and suppressed STAT1 translocation to the nucleus.	Indoleamine 2,3-dioxygenase, an immunomodulatory protein, is suppressed by EGCG via blocking of gamma-interferon-induced JAK-PKC-delta-STAT1 signaling in human oral cancer cells.	Cheng et al. (2010)
Formononetin (FN)	The C2C12 mouse myogenic progenitor cells	Decreased JAK1-STAT1 phosphorylation level.	FN treatment activates myogenic differentiation by increasing p38MAPK and decreasing JAK1-STAT1 phosphorylation levels.	Soundharrajan et al. (2019)
	The human colon carcinoma cell lines SW1116 and HCT116	Reduced p-STAT3 protein level.	FN suppresses cell proliferation and invasion by inhibition of cyclin D1 and MMP2/9 expression via p-STAT3 inactivation in colon carcinoma cells.	Wang et al. (2018)
	HuVecs	Inhibited the phosphorylation and the mRNA expression levels of JAK2 and STAT.	FN may be a new potential therapeutic compound for the treatment of vascular complications of diabetes.	Zhou et al. (2019)
	Human MM cell line U266 and human myeloma cell line RPMI 8226, athymic nu/nu female mice	Inhibited the cascade of STAT3 and STAT5 signals.	FN shows anticancer effect in MM.	Kim et al. (2018)
Genistein	Balb/c mice	Inhibited STAT6.	Modulates the Th1/Th2 reaction by inhibiting GATA-3 and STAT6 production while increasing T-bet production.	Gao et al. (2012)
	Murine J774 macrophages	Inhibited activation of STAT1.	Genistein inhibited LPS-induced STAT-1 expression.	Hämäläinen et al. (2007)
Malvidin	THP1 human monocytic cells	Suppressed STAT3 phosphorylation and nuclear translocation.	Malvidin inhibits the JAK-STAT pathway and blocks inflammation.	Baba et al. (2017)
α-Bromo-2′,3,4,4′-tetramethoxychalcone (α-Br-TMC)	Ba/F3 and Ba/F3-1*6 cells	α-Br-TMC inhibited JAK2 and STAT5 phosphorylation.	A-BR-TMC can be used in the treatment of STAT5-related malignancies.	Pinz et al. (2014)
3,4,2′,4′-tetrahydroxychalcone (Butein)	Human multiple myeloma (MM) U266	Inhibited the activation of JAK1/2.	Inhibition of tumor cell proliferation and reversal of chemotherapy resistance in multiple myeloma cells by blocking STAT3 activation.	Pandey et al. (2009)
Bavachin	IM9 cells, RPMI 8226 cells, and RPMI 1788 cells. Male BALB/c mice	Inhibited activation of STAT3.	Bavachin induces apoptosis by inhibiting the activation of NF-κB and STAT3 in multiple myeloma cell lines.	Takeda et al. (2018)
	Hep3B cells	Inhibited STAT3 promoter activity.	Bavachin treats inflammatory diseases by inhibiting the activation and phosphorylation of STAT3 induced by IL-6.	Lee et al. (2012)
Cardamonin	Female ICR mice	Inhibited the activation of STAT1 – 4.	Targets the production of IFN- and thereby suppresses the STAT pathway to mitigate inflammation.	Takahashi et al. (2011)
	The human colon cancer cell line HT-29 and SW-460, and C57BL/6 mice	Reduced the secretion of IL-1β and TNF-α, and inhibited the phosphorylation of STAT.	In the treatment of recurrent colitis and colitis-related tumors. ABU can inhibit cell viability and inflammatory cytokines of colorectal cancer cells *in vitro*.	Hou et al. (2019)
	CD133+GSCs	Inhibited the activation of STAT3 and the expression of downstream STAT3 genes, and prevented the migration of STAT3 to the nucleus and dimerization.	Cardamonin is a novel inhibitor of STAT3 and has the potential to be developed as a new anticancer agent targeting GSCs.	Wu et al. (2015)
5,7-Dihydroxyflavone	Human hepatocellular carcinoma (HepG2) and BALB/c female nude mice	Decreased the phosphorylation of STAT3.	The phosphorylation level of STAT3 was decreased, the antiapoptotic signal was weakened, and the growth of xenograft of HepG2 tumor was significantly inhibited.	Zhang et al. (2013)
Ginkgetin (GK)	C57BL/6 male mice and 3T3-L1	Inhibited STAT5 activity.	The inhibition of PPARγ and C/EBPα expression by *Ginkgo biloba* flavonoids was due to STAT5 inactivation at the initial stage of adipogenesis.	Cho et al. (2019)
	Human cancer cell lines HCT-116, DU-145, LNCap and PC-3	Mediated dephosphorylation of STAT3.	Ginkgetin blocks its entry into the nucleus, which in turn inhibits STAT3-mediated gene expression, thus inhibits the proliferation of DU-145 prostate cancer cells.	Jeon et al. (2015)
	Giant cell tumor samples	Inhibited STAT3 phosphorylation.	Ginkgetin significantly reduced survivin expression, which significantly inhibited tumor growth.	Xiong et al. (2016)
Casticin (CST)	786-O, HEL 299	Inhibited p-JAK1/2 and the expression of the phosphorSrc kinase	Casticin inhibits the JAK-STAT pathway in tongue squamous cell carcinoma, hypertriploid renal cell carcinoma, and oral squamous cell carcinoma.	Lee et al. (2019)
Kurarinone	The immortalized human keratinocyte cell line HaCaT and C57BL/6 mice or OT-II transgenic mice	Inhibitory phosphorylation of STAT1, STAT3, STAT5, and STAT6.	Suppresses JAK/STAT-dependent CD4+T-cell differentiation and improves chronic inflammatory skin diseases by inhibiting pro-inflammatory mediators, the JAK-STAT pathway, and the general immune response.	Kim et al. (2013)
Resveratrol	Sprague-dawley rats	Downregulated the p-JAK, p-STAT, and inflammatory cytokines.	Resveratrol protects hippocampal neurons from cerebral ischemia/reperfusion injury and alleviates cognitive dysfunction by regulating JAK-ERK-STAT signaling pathway.	Chang et al. (2018a)
	BTBR T+ Itpr3tf/J (BTBR) and C57BL/6 male mice	Decreased the expression levels of IL-6, TNF-α, IFN-γ, p-STAT1, and p-STAT3 (Tyr705).	Resveratrol can also inhibit JAK1-STAT3 in the brain tissue of autistic mice and treat neuroimmune dysfunction.	Ahmad et al. (2018)
	RAW 264.7 macrophages	Inhibited the transcriptional activity and phosphorylation of STAT1.	Resveratrol controls the inflammatory response of interferon-γ-activated macrophages.	Chung et al. (2011)
Caffeic acid (CA) and Caffeic acid phenethylester (CAPE)	The Caki-I human renal carcinoma and COS7 monkey kidney cell lines	Suppressed p-STAT3 and STAT3-inducible VEGF gene expression.	CA and CAPE suppress tumor angiogenesis by inhibiting the activity of STAT3 and the expression of HIF-1α and VEGF	Jung et al. (2007)
	SHRSP and WKY rats	Abolished the tyrosine phosphorylation of JAK2 and STAT1.	CA attenuated the proliferative response of SHRSP and WKY rat vascular smooth muscle cells to Ang stimulation by partially blocking the JAK-STAT signaling cascade and the RAS/RAF-1/ERK1/2 cascade.	Li et al. (2005)
	Male wistar rats (*Rattus norvegicus*)	Downregulated cerebral JAK2 expression, STAT3 phosphorylation, and SOCS3 protein expression.	CAPE can reduce oxidative stress and inflammation in the brain of rats, enhance antioxidant defense ability, and reverse the upregulation of Chromium (VI) on JAK2, STAT3 and SOCS3 in brain tissues.	Mahmoud and Abd El-Twab (2017)
Anwulignan	Human NSCLC cell lines A549, H1299, H1650, and H1975.	Inhibited the phosphorylation of STAT3 by directly targeting JAK1.	Anwulignan is a novel JAK1 inhibitor that may have therapeutic implications for NSCLC management.	Xie et al. (2021)
Honokiol	Human oral squamous cell carcinoma (OSCC) cell line	Reduced the levels of p-JAK2 and p-STAT3.	Honokiol suppressed the sphere formation and xenograft growth of oral CSC-like associated cells.	Huang et al. (2016)
	Breast cancer cells	Induced increase in tumor suppressor LKB1 led to dephosphorylation and inactivation of STAT3.	HNK inhibited breast tumorigenesis in mice in an LKB1-dependent manner.	Sengupta et al. (2017)
	Human leukemia cell lines (HEL and THP1)	Inhibited STAT3 transcription activity, reduced nuclear translocation of STAT3, and decreased STAT3 target gene expression.	HNK plays an anticancer role in acute myeloid leukemia by inhibiting STAT3 signaling.	Bi et al. (2015)
Curcumin	Sprague dawley male rats	Enhanced SOCS-1 expression.	The anti-inflammatory effect of curcumin is realized by enhancing SOCS-1 expression and inhibiting the JAK-STAT pathway.	Zhang et al. (2016)
	Human OS cell line (MG-63)	Inhibited p-JAK2 and p-STAT3.	Curcumin inhibits the JAK-STAT pathway to induce G0/G1 phase arrest and apoptosis, and inhibits the proliferation and migration of osteosarcoma cells.	Sun et al. (2019b)
Wedelolactone	HepG2, WiDr, A431, and A549 cells	Specifically inhibited TCPTP, the major phosphatase of STAT1, and prolonged IFN-γ-induced STAT1 phosphorylation.	Wedelolactone enhanced the antitumor effect of IFN-γ by inhibiting TCPTP-mediated STAT1 dephosphorylation.	Chen et al. (2013)

## Conclusion and Future Perspectives

Abnormal mutations in the JAK-STAT signaling pathway are closely related to the occurrence and development of malignant tumors, inflammation and autoimmune diseases. In this review, we wrote more than 20 kinds of polyphenolic phytochemicals and found that the mechanism of action of these polyphenol phytochemicals on the JAK-STAT signaling pathway is not completely the same. Some compounds can directly act on the JAK-STAT pathway, such as Luteolin, KPG, Butein, Casticin, Genistein, Silibinin, Kurarinone, 5,7-Dihydroxyflavone, Ginkgetin, Capillarisin, Bavachin, α-Br-TMC, Formononetin, Anwulignan, Capsaicin, Malvidin, these compounds can directly affect the JAK-STAT signaling pathway in three ways: 1) inhibit the phosphorylation of JAK and (or) STAT 2) inhibit the activation and expression of JAK and (or) STAT 3) interfere with the movement and nuclear translocation of STAT, and affect the regulation of STAT target genes. Other compounds have different mechanisms of action in different diseases, such as Apigenin, Quercetin, Hesperidin, TAHP, Cardamonin, Resveratrol, Caffeic acid phenethylester, Honokiol, and Curcumin. They can not only directly act on the JAK-STAT signal pathway through the above methods, but also indirectly regulate the JAK-STAT pathway through the following methods: 1) inhibit the secretion of interleukins, interferons, tumor necrosis factor and other cytokines 2) interfere with the process by which the JAK-STAT pathway is activated by cytokines 3) indirectly regulate the JAK-STAT signaling pathway by regulating endogenous inhibitors in the pathway, such as SOCS.

According to the chemical structure, we divided the 26 phytochemicals in this article into flavonoids, phenolic acids, stilbenes, lignans and some compounds with complex parent nuclei, and classified the flavonoids in more detail. We found that some compounds have similar pharmacological effects, which may be related to their chemical structure. Flavones are compounds with 2-phenyl-chromone as parent nuclei, including Apigenin and Luteolin. We found that flavones represented by Apigenin and Luteolin can directly inhibit the phosphorylation of STAT3 in breast cancer cells, which may be related to the structure of flavones. Flavonols are derivatives of 2-phenylchrominones-3-alcohols, including kaempferol, quercetin. Both KPG and Quercetin inhibit p-JAK2 and p-STAT3 in inflammatory diseases. Flavanones have 2-phenyl-2,3-Chromanone basic nucleus, Flavanones include Hesperidin, Liquiritin. Hesperidin and Silibinin can interfere in different diseases by affecting STAT3 expression and phosphorylation. Flavanonols have 2-phenyl-2,3-Chromanone-3-alcohol nucleus. The mother nucleus structure of Anthocyanidins is 2-phenylbenzopyrylium salts or flavylium salts and Malvidin is one of the more common anthocyanins. Malvidin significantly down-regulates the expression of JAK2 and STAT3 phosphorylated forms, and inhibits the STAT3 nuclear translocation. The skeleton of Chalcones is formed by the opening of the C ring of flavanonols. The numbering of the carbon atoms of the nucleus is different from other types of flavonoids. The carbon of the A ring is 1′–6′, and the carbon of the B ring is 1–6. Among the four Chalcones, Butein, Bavachin and Cardamonin can play a role in multiple myeloma and other diseases by inhibiting the activation and expression of STAT3. Lignans are natural products formed from two structures with phenylpropane skeletons connected by the β, β′, or 8, 8′-carbons. Anwulignan and Honokiol can down-regulate p-STAT3 in various cancers (NSCLC, breast cancer, oral cancer, and myeloid leukemia). Phenolic acid compounds refer to aromatic carboxylic acid compounds substituted with multiple phenolic hydroxyl groups on a benzene ring. They are plant secondary metabolites. Phenolic acids can be divided into two categories according to their carbon skeleton structure: Hydroxybenzoic acid and hydroxycinnamic acids. There are two other types that are formed by condensation: chlorogenic acid is a combination of caffeic acid and quinic acid through an ester bond, and salvianolic acid is a polymer of simple phenolic acid. Among the members of the STATs family of proteins, STAT3 plays an important role in cell proliferation, inflammation, tumorigenesis and other vital activities and is most frequently activated and mutated in the occurrence and development of tumors. It was found that most polyphenol phytochemicals have an effect on STAT3.

There are currently many studies on inhibitors of JAK kinase, such as tofacitinib and ruxolitinib, which have been approved by the United States Food and Drug Administration (FDA), which have a good inhibitory effect on some lymphoma tumors. However, at higher doses, they exhibit “pan-JAK” inhibitory effects, resulting in off-target effects, causing infection, malignant tumors, venous thromboembolism (VTE), dyslipidemia, gastrointestinal perforation and other adverse reactions (Reddy and Cohen, 2020). For example, on February 25, 2019, the first JAK inhibitor tofacitinib on the market found that high-dose tofacitinib in patients with rheumatoid arthritis could lead to the risk of pulmonary thrombosis and death. On August 28, 2019, the FDA also required the drug label to add a black box warning of “thrombosis and death risk” (Kaufmann et al., 2018). At this point, polyphenolic compounds that also target JAK such as KPG, EGCG, Butein, and Casticin have been found to have less adverse effects and toxicity currently. STATs family proteins are highly active in malignant tumors and other diseases, but the development of STATs inhibitors has always been a major problem due to their bioavailability and the difficulty of improving the selectivity *in vivo*. Therefore, most drugs targeting STATs are still in the preclinical stage. Apigenin, Luteolin, Silibinin and other compounds can specifically inhibit STAT. Honokiol inhibits both constitutive and inducible STAT3 activation and reduces the mRNA expression of STAT3 target genes (Bcl-2, cyclin D1, and survival) in myeloid leukemia cells in a concentration-dependent manner. Formononetin (FN) can inhibit both STAT3 and STAT5 in human MM cells. These compounds can make up for the current lack of STAT inhibitors.

But compared with polyphenolic phytochemicals, JAK inhibitors also have their advantages. In the treatment of RA, JAK inhibitors have a unique mechanism of action, which has the advantages of rapid onset, long-lasting efficacy, and no risk of secondary failure, which are different from biological agents (Buhl et al., 2021). For example, in a long-term clinical trial (NCT00413699), tofacitinib showed its stable efficacy for up to 9.5 years. Polyphenols contain a large amount of phenolic hydroxyl groups, many of them are poorly water-soluble due to the spatial structure, which may lead to huge differences in the results of *in vivo* and *in vitro* models. Part of the reason is that the metabolism of polyphenols in the body is very complicated. For example, many insoluble components can be metabolized by gut microbiota. This is especially obvious in phenolic acid compounds, due to the substitution of more phenolic hydroxyl groups in phenolic acid compounds’ structure, the structure is not stable, and it is easy to denature under the action of acidic, alkaline environment, unsuitable temperature and enzymes. There is abundant experimental data on the intervention of Flavonoids and less data on the effect of other types of polyphenol phytochemicals on the pathway. The limitations of many phytochemicals include low water solubility, poor absorption and bioavailability, which may limit their effects in clinical treatments. Unlike drugs that can be administered in specific doses and at specific times in a particular situation, Phytochemicals are often ingested at random intervals and concentrations in people’s diets. Achieving adequate phytochemical concentrations and bioavailability, by ingesting foods at concentrations similar to those of the drug in the study environment, is a challenge (Bose et al., 2020). These impressive findings confirm the importance and necessity of phytochemicals in clinical trials.

More than 20 compounds have different pharmacological effects in the JAK-STAT signaling pathway. However, we believe that curcumin may be the most promising potential treatment in the future. First of all, curcumin has been proven effective in animal and cell experimental models of a variety of diseases. In addition to the two mechanisms listed above, it is also effective in intestinal inflammation (Zhang et al., 2016), Papillary Thyroid Cancer (Khan et al., 2020), triple-negative breast cancer (Bonaccorsi et al., 2019), Small Cell Lung Cancer (Yang et al., 2012) and other diseases have been proven to have a therapeutic effect by acting on the JAK-STAT pathway. For example, in the Papillary Thyroid Cancer cell line, by inhibiting JAK/STAT3 activity and inhibiting anti-apoptotic genes and inducing pro-apoptotic genes, curcumin and cisplatin synergistically enhance cytotoxicity, and also through down-regulation of matrix metalloproteinases and inhibition of colony formation to inhibit the migration of PTC cells. In addition, curcumin can not only directly act on the JAK-STAT pathway, but also indirectly play a role by inhibiting the secretion of cytokines and enhancing the negative regulatory factors in the cells.

Owing to the nature of the JAK-STAT pathway and its potential cross-epitope with multiple alternative pathways, the long-term efficacy of adaptive targeted therapies based on monotherapy is limited. At the same time, phytochemicals have shown good potency in the treatment of cancer, diabetes, Alzheimer’s disease, and other diseases (Batra et al., 2019). Polyphenols can inhibit abnormal activation by acting on multiple steps in the JAK-STAT pathway. For example, Curcumin inhibits both the constitutive and IL-6-induced STAT3 phosphorylation and IFN-induced STAT1 phosphorylation. The multi-activity and multi-targeting of phytochemicals and their mitigation of the toxic and side effects of chemotherapeutic agents have shown better clinical effects. For example, Compared with silibinin alone, JAK1 inhibitors combined with silibinin also lead to complete reduction of STAT3 phosphorylation at Tyr705, activation of caspase, and apoptosis of prostate cancer cells. Therefore, it may be worthwhile to further explore the use of JAK/STAT inhibitors in combination with polyphenolic phytochemicals.

## Glossary

Ang II: angiotensin II
CA: caffeic acid
CAPE: caffeic acid phenethy lester
C/EBPα: CCAAT/ enhancer binding protein α
EGCG: Epigallocatechin-3-gallate
EAE: experimental autoimmune encephalomyelitis
EGF: epidermal growth factor
EMT: epithelial–mesenchymal transition
FN: Formononetin
GH: growth hormone
GK: Ginkgetin
GM-CSF: granulocyte-macrophage colony-stimulating factor
GOF: Gain-of-function
HMEC-1: human microvascular endothelial cells
IFNs: interferons
IL: interleukins
iNOS: inducible nitric oxide synthase
JAK: janus kinase
JH: JAK homology
LKB1: Liver kinase B1
LOF: Loss-of-function
LPS: lipopolysaccharide
MMP: matrix metalloproteinase
NLF: Nasal lavage fluid
NSCLC: non-small-cell lung carcinoma
OVA: ovalbumin
PDGF: platelet-derived growth factor
PIAS: protein inhibitors of activated STATs
p-JAK2: phosphorylated JAK2
PPARγ: peroxisomal proliferation-activated receptor γ
PSA: psoriatic arthritis
p-STAT3: phosphorylated STAT3
PTPs: protein tyrosine phosphatases
RA: rheumatoid arthritis
SH2: the Src-homology type 2
SHP-1: Src homology region 2 domain-containing phosphatase 1
SOCS: suppressor of cytokine signaling
STAT: signal transducer and activator of transcription
TAHP: 5,7,3ʹ-triacetyl hesperetin
T-bet: T-box transcription factor
TCPTP: T-cell protein tyrosine phosphatase
VEGF: Vascular Endothelial Growth Factor
